# Caloric restriction, physical exercise, and CB1 receptor blockade as an efficient combined strategy for bodyweight control and cardiometabolic status improvement in male rats

**DOI:** 10.1038/s41598-021-83709-9

**Published:** 2021-02-19

**Authors:** Luisa M. Lopez Trinidad, Rosario Martinez, Garyfallia Kapravelou, Milagros Galisteo, Pilar Aranda, Jesus M. Porres, Maria Lopez-Jurado

**Affiliations:** 1 Department of Physiology, Institute of Nutrition and Food Technology (INyTA), Centre for Biomedical Research, Centre for Research in Sport and Health (IMUDS), Universidad de Granada, Avda. del Conocimiento S/N. Armilla (18100), Granada, Spain; 2 Department of Pharmacology, School of Pharmacy, Biohealth Research Institute, Centre for Biomedical Research, Universidad de Granada, Granada, Spain

**Keywords:** Weight management, Metabolism, Endocrine system and metabolic diseases

## Abstract

Obesity is critically associated with the development of insulin resistance and related cardiovascular and kidney diseases. Several strategies for weight loss have been developed but most of them exhibit a post-intervention rebound effect. Here, we aimed to design combined weight-loss strategies of caloric restriction, physical exercise, and administration of a CB1 receptor blocker to inhibit food intake that also accomplish the objectives of lost-weight maintenance and improvement of cardiovascular and renal function. Diet-induced obesity (DIO) was generated in Sprague Dawley rats for 12 weeks to test the effects of single or combined strategies (i.e. caloric restriction, mixed training protocol, and/or administration of appetite suppressant) on caloric intake, body weight, cardiovascular and renal functionality resulting from a weight-loss intervention period of 3 weeks followed by 6 weeks of weight maintenance. Consumption of a high-fat diet (HFD) caused a significant increase in body weight (5th week of the experimental period) and led to the development of insulin resistance, cardiovascular, and renal alterations. The different interventions tested, resulted in a significant body weight loss and improved glucose metabolism, aerobic capacity, electrocardiographic parameters, vascular expression of adhesion molecules and inflammatory mediators, and renal functionality, reaching values similar to the control normocaloric group or even improving them. Successful maintenance of lost weight was achieved along a 6-week maintenance period in addition to adequate health status. In conclusion, the weight-loss and maintenance intervention strategies tested were efficient at reversing the obesity-related alterations in body weight, glucose metabolism, aerobic capacity, cardiovascular and renal functionality. The beneficial action was very consistent for caloric restriction and physical exercise, whereas administration of a CB1 receptor blocker complemented the effects of the prior interventions in some parameters like body weight or aerobic capacity, and showed specific actions in renal status, increasing glomerular filtration rate and diuresis. Overall, the novelty of our study relies on the easy implementation of combined strategies for effective weight management that resulted in significant health benefits.

## Introduction

Overweight and obesity are defined as abnormal or excessive fat accumulation that increases the risk to develop multiple pathologies. They lead to adverse metabolic effects on blood pressure, cholesterol, triglycerides, and insulin resistance. This compilation of factors is known as metabolic syndrome (MetS)^1^, which is directly related to cardiovascular disease and the alteration in other vital functions, such as the renal function^2^. Overweight, obesity and its related diseases are largely preventable^3^. In this regard, a comprehensive understanding of MetS may be important for the adequate planning of prevention strategies. Since its components are all reversible, early diagnosis and lifestyle intervention strategies of MetS offer an effective treatment approach, primarily targeting weight management.

The control and maintenance of the body weight at a stable level are achieved when there is a balance between food intake and energy expenditure. A complex physiological control system is involved in the maintenance of the energy balance and includes afferent signals from the periphery regarding the state of energy stores, as well as efferent signals affecting energy intake and expenditure^4^. This regulatory system includes multiple interactions between the gastrointestinal tract, adipose tissue and central nervous system, and is influenced by behavioral, sensorial, autonomic, nutritional and endocrine mechanisms^5^.

The first step in the treatment of obesity is focused on losing the extra-weight and ameliorate the related metabolic alterations. Another important issue for subjects who complete a weight loss program is to avoid the post-intervention rebound effect. The bodyweight regain usually takes place right after the end of weight loss intervention as weight loss programs are just transient^6^. A multidisciplinary approach is required, including lifestyle modifications^7^ and, in some cases, the reinforcement with pharmacological treatment. Related to lifestyle interventions there are two core aspects to correct an altered energy balance: diet and physical exercise. Obesity development is often favored by the consumption of unbalanced and hypercaloric diets, so the caloric restriction of a balanced diet providing adequate amounts of nutrients is highly recommended. On the other hand, physical activity plays an essential role in the prevention and treatment of obesity. It contributes to generating a negative energy balance, thus facilitating weight loss and avoiding the rebound effect and subsequent body weight regain^8^. It is well established that different training protocols induce changes in a variety of molecular mechanisms involved in numerous intracellular pathways related to glucose and lipid metabolism, inflammation, or antioxidant status^9^.

In addition to lifestyle modifications, the prescription of an appropriate pharmacological agent is sometimes recommended. The endocannabinoid system is comprised by cannabinoid receptors 1 and 2 (CB1 and CB2), the two endocannabinoids anandamide and 2-arachidonoylglycerol, and endocannabinoid anabolic and catabolic enzymes^10^. The endocannabinoid system (ECS) plays a critical role in obesity development in both central and peripheral functions related to energy metabolism^11^. At the central level, endocannabinoids act as retrograde neuromodulators of synaptic plasticity, and participate in many physiological processes including pain regulation, learning and memory, appetite and food intake, lipogenesis, and cravings^12,13^. At the peripheral level, endocannabinoids exert a tonic action on lipogenesis and fat accumulation. Therefore, CB1 blockade may result in a food intake-independent decrease in fat mass through lipolysis^14^. In fact, CB1 blockade has been shown to be effective in ameliorating obesity and related metabolic disorders^15^. In addition, the endocannabinoid 2-AG prevent myotube formation in a manner antagonized by CB1 knockdown and by CB1 antagonists, which per se, instead, stimulate differentiation^16^. Moreover, antagonism of CB1 receptor reduced human satellite cell proliferation and enhanced the formation of myotubes representing an adjuvant therapy of muscle dystrophies^17^.

In recent years, the discovery of an expanded endocannabinoid system, the endocannabinoidome, which includes several mediators that are biochemically related to the endocannabinoids, and their receptors and metabolic enzymes, has corroborated its complexity and expanded the potential for developing new therapeutic strategies to treat multiple related pathologies including neurological, inflammatory or metabolic alterations^10^.

Given the above mentioned, we hypothesized that our specific combined strategy of lifestyle and pharmacological interventions could provide interesting benefits in the treatment of obesity and its related cardiovascular and renal alterations. Therefore, this study aimed to design these new strategies and test them in an experimental model of DIO. Specifically, we sought to (1) test the effects on body weight, physical performance, glycaemic and lipid profile, and different parameters related to cardiovascular and renal health, of a combined program with caloric restriction, mixed training exercise protocol, and pharmacological treatment with the appetite suppressant AM251, a well-known research tool that effectively blocks CB1 receptors and exhibits a strong food intake inhibition action combined with increased basal metabolic rate and decreased plasma levels of glucose and LDL-cholesterol. (2) study the synergies taking place between the three above-mentioned strategies both on body weight loss and in the maintenance of weight and metabolic, cardiovascular and renal benefits acquired.

## Results

### Combined weight control strategies efficiently achieve weight loss and prevent body weight rebound after treatment

**Figure 1:**
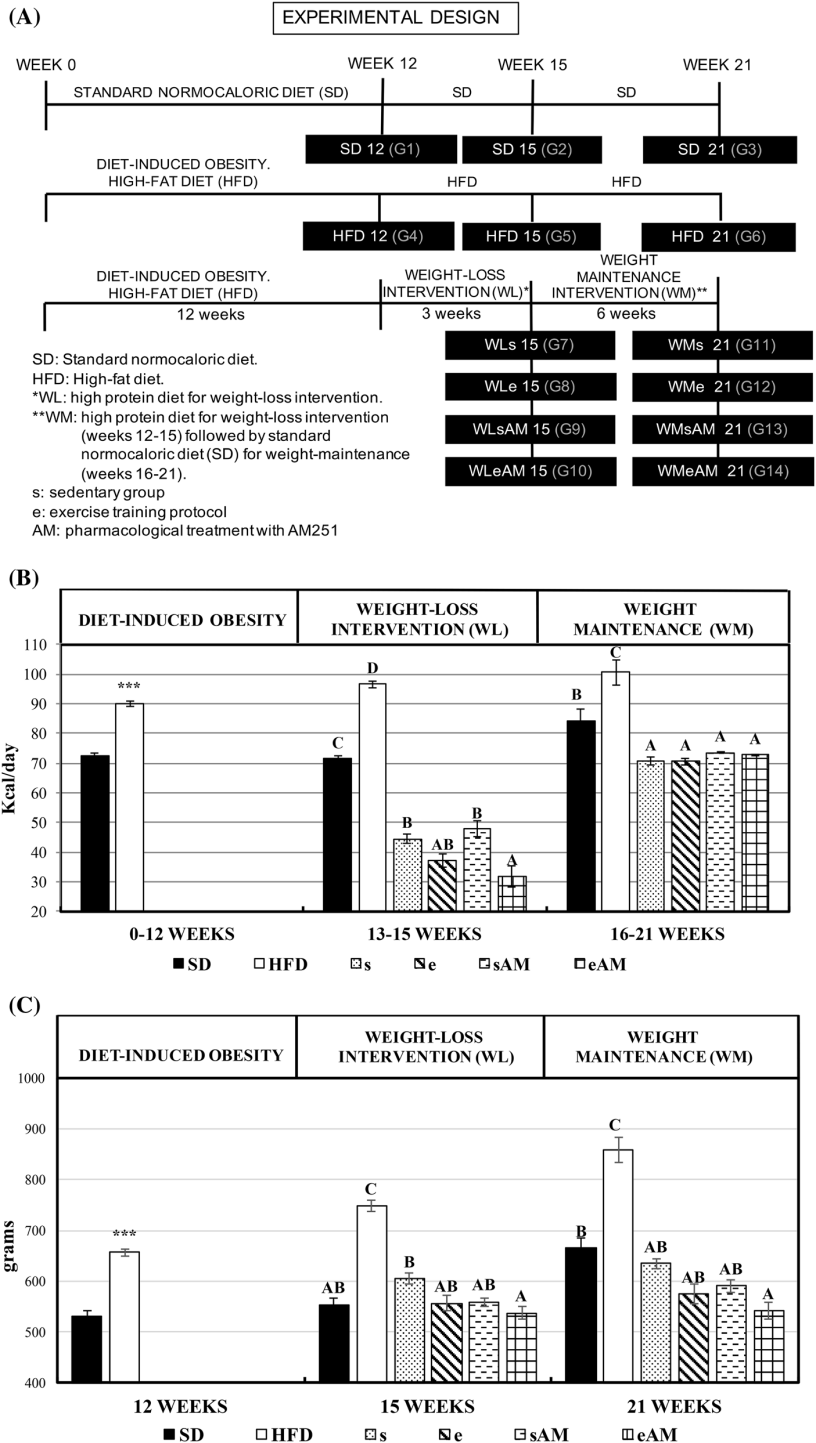

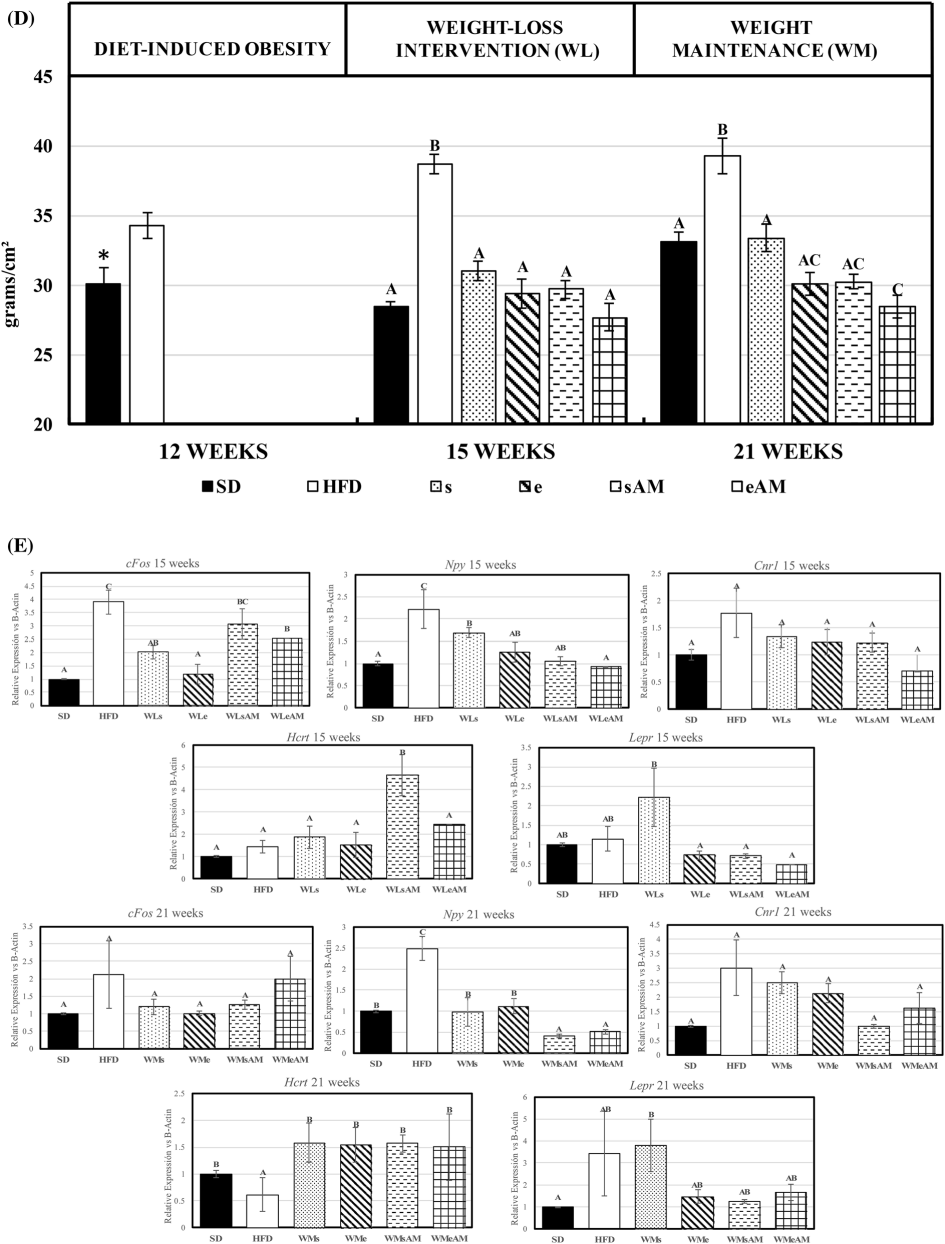
Effects of DIO and weight control interventions on caloric intake, body weight, body weight/femur length ratio, and hypothalamic gene expression of transcripts involved in the central regulation of food intake and energy balance. Six control experiments were carried out during 21 weeks using a standard rat chow diet (SD12, SD15, and SD21) or a high-fat diet to induce obesity (HFD12, HFD15, and HFD21). For intervention trials, rats were divided into 8 groups that were fed the hypercaloric diet to induce obesity for 12 weeks, followed by three weeks of intervention with a high protein diet for weight loss (WL15) combined or not with the training protocol (e or s, respectively) and the pharmacological treatment with CB1 receptor blocker AM251 (AM). The intervention period was followed by an additional 6-week weight-maintenance period of dietary treatment with a standard rat chow diet (WM21) combined or not with the training protocol (e or s, respectively) and the pharmacological treatment (AM) to maintain the weight lost during the previous intervention period of three weeks. (**A**) Experimental design, (**B**) average daily caloric intake (kcal/day) along the different experimental stages (DIO, weight-loss intervention, and weight-maintenance), (**C**) average body weight at the end of the different experimental stages, (**D**) bodyweight/ femur length ratio (g/cm^2^) at the end of the different experimental stages, (**E**) hypothalamic gene expression at the end of the weight-loss intervention and weight-maintenance stages. Results are means of eight rats ± SEM depicted by vertical bars. ***P < 0.001 in t-test (12 weeks); A,B,C means with different letters are significantly different (ANOVA treatment, P < 0.05; 15 and 21 weeks). *cfos* c-Fos, *npy* neuropeptide Y, *lepr* leptin receptor, *hcrt* orexin A, *cnr1* cannabinoid receptor.

The effects of DIO and different weight-loss and weight-maintenance interventions on daily caloric intake and body weight at the end of the different experimental stages are presented in Fig. 1A–C.

Throughout the experimental period, and due to the higher caloric content of HFD, a significantly higher caloric intake was exhibited by HFD-treated rats when compared to their normocaloric controls. Likewise, higher body weight was gained by the former animals. During the weight-loss intervention period from week 12–15 of the experimental period, the individual action of caloric restriction, physical exercise, and AM251 administration caused a decrease in caloric intake that led to a concomitant decrease in body weight of treated rats. However, stronger effects were observed when the three interventions were combined. Along the weight-maintenance stage, a stabilization of caloric intake and maintenance of bodyweight among the different intervention groups were achieved below the values shown by the SD and HFD controls. Besides, the training protocol and the administration of the appetite suppressant played a role to avoid the rebound effect on body weight. These beneficial actions on body weight were reflected on a similar trend in body weight/femur length ratio at the different experimental stages, especially when exercise was combined with AM251 administration. (Fig. 1C). Such ratio has been considered representative of the body mass index and adequate to establish the degree of obesity and evolution of rats in a long experimental period.

The expression of c-fos and Npy increased in animals fed the HFD on weeks 15 and 21 compared to the SD group, and decreased as a result of bodyweight loss. Such decrease was only observable in Npy during the lost-weight maintenance stage (Fig. 1D). The administration of AM251 showed an inhibitory action on Npy and CB1 receptor.

### Weight-loss interventions cause a significant improvement in aerobic capacity and glycemic profile

**Figure 2:**
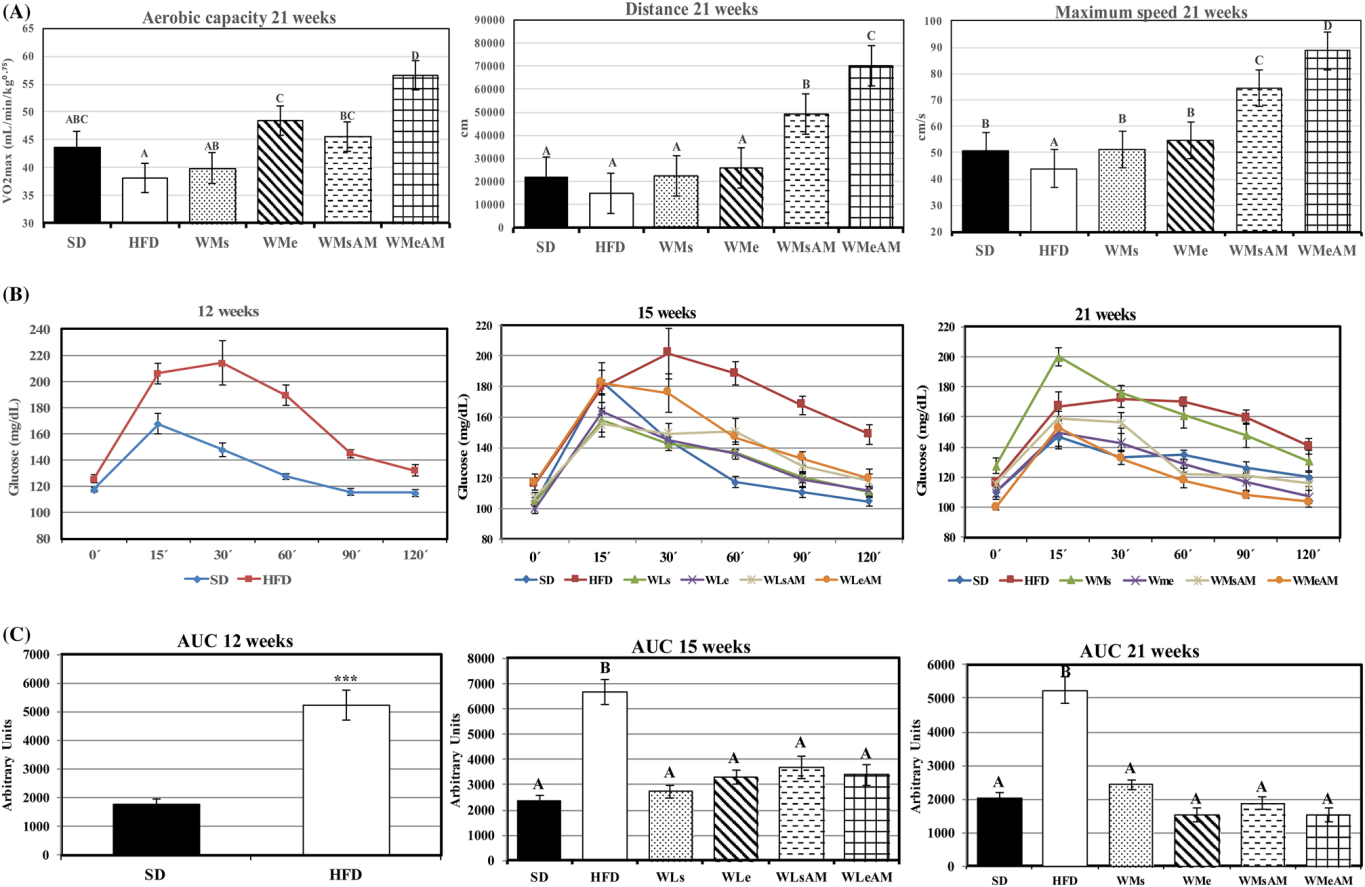
Effects of DIO and weight control interventions on aerobic capacity and glucose metabolism. (**A**) maximal oxygen consumption, maximum speed, and distance run during an incremental test were measured as markers of long term effects after 21 weeks of the experimental period, (**B**,**C)** glycemic profile and area under the curve after an oral glucose overload measured at 12, 15 or 21 weeks of the experimental period. Results are means of eight rats ± SEM depicted by vertical bars. *P < 0.05 in t-test (12 weeks); A,B,C means with different letters are significantly different (ANOVA treatment, P < 0.05; 15 and 21 weeks). *AUC* area under the curve (arbitrary units), *SD* normocaloric standard diet group, *HFD* HFD-treated group. Weight-loss interventions (WL) on weeks 13–15: *WLs* obese rats treated with caloric restriction and no exercise, *WLe* obese rats treated with caloric restriction in combination with physical exercise, *WLsAM* obese rats treated with caloric restriction in combination with AM251 administration and no exercise, *WLeAM* obese rats treated with caloric restriction in combination with physical exercise and AM251 administration. Weight-maintenance interventions (WM) on weeks 16–21: *WMs* rats treated with diet SD and no exercise, *WMe* rats treated with diet SD in combination with physical exercise, *WMsAM* rats treated with diet SD in combination with AM251 administration and no exercise, *WMeAM* rats treated with diet SD in combination with physical exercise and AM251 administration.

Overall, DIO caused a significant decrease in aerobic capacity and physical fitness parameters (distance and maximum speed) compared to the SD group at the end of week 21 of experimental period (Fig. 2A). The interventions assayed for bodyweight control were efficient at increasing the above-mentioned parameters *vs* the HFD groups and the combined effects of caloric restriction and physical exercise (WMe) were of especial relevance. Moreover, such effects were potentiated by AM251 administration that resulted in the highest values for all three parameters in the WMeAM group (P < 0.05). Glycemic profile and AUC after an oral glucose overload carried out at the end of every experimental stage are shown in Fig. 2B,C. DIO resulted in higher AUC, a higher postprandial blood glucose peak after 30 min of administration, and higher values of glycemia during a more prolonged period after glucose overload, when compared to the SD group. These data point to a situation of insulin resistance, which was reversed by the weight-loss/maintenance interventions assayed.

### Cardiovascular functionality is significantly improved by weight-loss and remains stable during the maintenance period

The obesity-related increase in body weight led to a hypertrophied heart whereas weight-loss interventions decreased heart weight to similar values as those found in the SD group in all experimental stages (Table 1). Similarly, ventricular electrocardiographic parameters were strongly affected in the HFD groups that showed a significant increase in the amplitudes of QRS complex and T wave along the experimental period. The increased amplitudes were reversed upon body weight loss achieved by the different interventions assayed, reaching values that were equal or even inferior to the SD group. About the QTc interval, it was increased by obesity and tended to decrease by caloric restriction and physical exercise except for group WMeAM on week 21. No relevant changes were observed in any of the parameters related to atrial functionality. Plasma activity of CK-MB was increased in animals that consumed the HFD on weeks 15 and 21 and decreased by the different weight-loss interventions in these experimental stages (Table 2).

**Table 1:** Effects of DIO and weight control interventions on parameters of heart functionality.

	Bodyweight (g)	Heart weight (g)	Heart rate (bpm)	P wavelength (s)	P wave amplitude (mV)	PR interval (s)	QRS length (s)	QRS amplitude (mV)	T wave length (s)	T wave amplitude (mV)	QTc interval (s)
**12 weeks**											
SD	516.9	1.53	293.7	0.019	0.105	0.027	0.019	1.35	0.023	0.184	0.098
HFD	648.7***	1.65	290.0	0.020	0.134*	0.025	0.020	1.69*	0.027	0.208*	0.106
SEM	19.7	0.045	7.3	0.002	0.010	0.002	0.003	0.075	0.001	0.035	0.056
**15 weeks**											
SD	503.3b	1.41a	263.5a	0.029b	0.112a	0.059b	0.021a	1.15a	0.031a	0.220b	0.103a
HFD	704.1a	1.96b	242.4a	0.022b	0.066a	0.055b	0.021a	1.76b	0.040c	0.308b	0.128b
WLs	566.7c	1.54a	265.3a	0.024ab	0.117a	0.064b	0.021a	0.911a	0.042ab	0.143a	0.121ab
WLe	552.5bc	1.50a	260.9a	0.020b	0.130a	0.030a	0.021a	1.15a	0.041bc	0.192a	0.125b
WLsAM	544.2bc	1.55a	268.0a	0.025ab	0.119a	0.065b	0.020a	0.885a	0.064d	0.188a	0.171c
WLeAM	511.5bc	1.45a	254.4a	0.019b	0.106a	0.059b	0.020a	1.12a	0.041bc	0.161a	0.123b
SEM	21.2	0.09	7.8	0.002	0.041	0.003	0.001	0.105	0.002	0.015	0.006
**21 weeks**											
SD	631.7a	1.69a	267.9a	0.021ab	0.089a	0.061ab	0.023ab	1.64b	0.042a	0.176a	0.134ab
HFD	803.8b	2.02b	274.5a	0.023b	0.129b	0.063ab	0.021a	1.97c	0.049a	0.291b	0.151b
WMs	634.2a	1.73a	263.7a	0.020ab	0.103ab	0.061ab	0.021a	1.09a	0.039a	0.203a	0.124a
WMe	574.4a	1.64a	255.9a	0.020ab	0.127a	0.067b	0.027b	1.14a	0.039a	0.206a	0.135ab
WMsAM	546.4a	1.72a	257.1a	0.022b	0.076a	0.057a	0.022ab	1.32a	0.045a	0.186a	0.134ab
WMeAM	590.9a	1.64a	273.9a	0.017c	0.095b	0.054a	0.021a	1.28a	0.049a	0.211a	0.152b
SEM	41.8	0.093	6.5	0.001	0.010	0.002	0.002	0.102	0.004	0.011	0.008

*SD* standard rat chow diet, *HFD* hypercaloric diet for dietary induction of obesity, *WLs* high protein weight-loss intervention diet with a sedentary lifestyle (weeks 12–15), *WLe* high protein weight-loss intervention diet with training protocol, *WLsAM* high protein weight-loss intervention diet with a sedentary lifestyle and pharmacological treatment with AM251, *WLeAM* high protein weight-loss intervention diet with training protocol and pharmacological treatment with AM251. *WMs* high protein weight-loss intervention diet with a sedentary lifestyle (weeks 12–15) followed by weight-maintenance stage (weeks 15–21) with SD dietary treatment and sedentary lifestyle, *WMe* high protein weight-loss intervention diet with training protocol followed by weight-maintenance stage with SD dietary treatment and training protocol, *WMsAM* high protein weight-loss intervention diet with a sedentary lifestyle and pharmacological treatment with AM251followed by weight-maintenance stage with SD dietary treatment, sedentary lifestyle, and pharmacological treatment with AM251, *WMeAM* high protein weight-loss intervention diet with training protocol and pharmacological treatment with AM251 followed by weight-maintenance stage with SD dietary treatment, training protocol, and pharmacological treatment with AM251, *Bpm* bits per minute, *QTc* corrected QT interval. Results are means of 8 rats. *SEM* standard error of the mean. *P < 0.05 in t-test (12 weeks); a, b, c, means within the same column with different letters are significantly different (ANOVA treatment, P < 0.05; 15 and 21 weeks).

**Table 2:** Effects of DIO and weight control interventions on plasma parameters of heart and kidney functionality.

	CK-MB (U/dL)	ACE (U/dL)	Total Proteins (g/dL)	Albumin (g/dL)	Urea (mg/dL)	Uric Acid (mg/dL)	Creatinine (mg/dL)	Phosphorus (mg/dL)	Calcium (mg/dL)
**12 weeks**									
SD	395.6	69.1	5.87	2.66	28.0	0.95	0.57	6.08	6.41
HFD	292.4	81.8	6.17*	2.92	32.7*	0.69	0.11	5.95	9.80***
SEM	0.16	0.67	0.14	0.15	0.16	0.16	0.08	0.41	0.67
**15 weeks**									
SD	358.9a	73.4a	6.22c	3.33b	28.2a	0.94ab	0.71bc	6.13b	6.33a
HFD	987.0b	73.6a	6.51c	3.08ab	31.0a	0.90ab	0.15a	5.40ab	10.1b
WLs	335.6a	57.5b	5.57a	2.88ab	28.0a	0.72a	0.49abc	4.93a	6.59a
WLe	362.1a	77.3a	5.85ab	2.86ab	29.7a	0.92ab	0.81c	5.45ab	6.73a
WLsAM	213.6a	72.3a	5.34a	2.61a	34.0a	1.02ab	0.40ab	5.41ab	10.8b
WLeAM	196.1a	79.8a	5.42a	2.73ab	26.2a	1.19b	0.40ab	6.16b	11.0b
SEM	154.0	9.42	0.18	0.21	3.10	0.15	0.13	0.30	0.86
**21 weeks**									
SD	166.7a	65.0a	6.39a	2.92ab	32.3b	0.78a	0.054a	6.28a	8.68a
HFD	571.7b	70.8b	6.07a	2.94ab	24.3ab	1.07a	0.046a	5.93a	9.51ab
WMs	206.4a	85.7bc	6.24a	3.09ab	24.0a	1.29ab	0.47a	5.59a	7.86a
WMe	207.6a	96.7d	6.09a	3.37b	26.6ab	0.86a	0.55a	5.99a	7.79a
WMsAM	283.0a	72.5b	6.00a	2.60a	25.6ab	1.29ab	0.54a	5.85a	11.1b
WMeAM	254.0a	75.4b	5.74a	3.09ab	40.6c	1.24ab	0.55a	5.63a	10.1ab
SEM	307.4	11.8	0.23	0.22	2.70	0.24	0.67	0.57	0.99

*SD* standard rat chow diet, *HFD* hypercaloric diet for dietary induction of obesity, *WLs* high protein weight-loss intervention diet with a sedentary lifestyle (weeks 12–15), *WLe* high protein weight-loss intervention diet with training protocol, *WLsAM* high protein weight-loss intervention diet with a sedentary lifestyle and pharmacological treatment with AM251, *WLeAM* high protein weight-loss intervention diet with training protocol and pharmacological treatment with AM251. *WMs* high protein weight-loss intervention diet with a sedentary lifestyle (weeks 12–15) followed by weight-maintenance stage (weeks 15–21) with SD dietary treatment and sedentary lifestyle, *WMe* high protein weight-loss intervention diet with training protocol followed by weight-maintenance stage with SD dietary treatment and training protocol, *WMsAM* high protein weight-loss intervention diet with a sedentary lifestyle and pharmacological treatment with AM251 followed by weight-maintenance stage with SD dietary treatment, sedentary lifestyle, and pharmacological treatment with AM251, *WMeAM* high protein weight-loss intervention diet with training protocol and pharmacological treatment with AM251 followed by weight-maintenance stage with SD dietary treatment, training protocol, and pharmacological treatment with AM251. *CK-MB* creatine kinase MB. Results are means of 8 rats. SEM, standard error of the mean. *P < 0.05, ***P < 0.001 in t-test (12 weeks); a, b, c, means within the same column with different letters are significantly different (ANOVA treatment, P < 0.05; 15 and 21 weeks).

**Figure 3:**
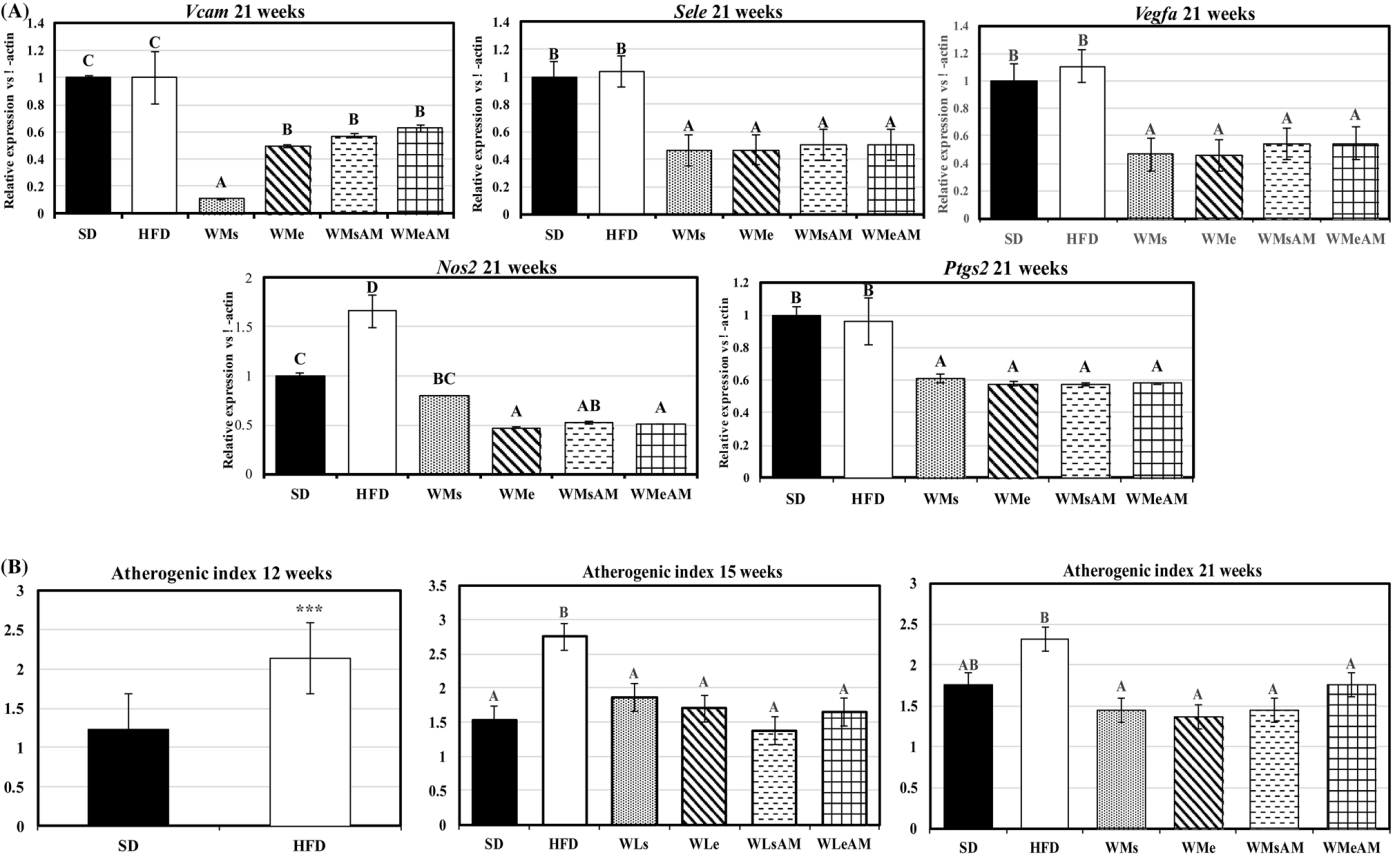
Effects of DIO and weight control interventions on vascular damage measured in the aorta and plasma. (**A**) gene expression of transcripts coding for vascular adhesion molecules and inflammation markers at the end of the 21-week experimental period, (**B**) plasma atherogenic index at the end of the different experimental stages. Results are means of eight rats ± SEM depicted by vertical bars. *P < 0.05 in t-test (12 weeks); A,B,C means with different letters are significantly different (ANOVA treatment, P < 0.05; 15 and 21 weeks). *Vcam* vascular cell adhesion molecule 1, *Vegfa* Vascular endothelial growth factor A, *Sele* Selectin E, *Nos2* Nitric oxide synthase 2, *Ptgs2* Prostaglandin-endoperoxide synthase 2. Atherogenic index, (total-cholesterol (mg/dL)/HDL-cholesterol (mg/dL). *SD* normocaloric standard diet group, *HFD* HFD-treated group. Weight loss interventions (WL) on weeks 13–15: *WLs* obese rats treated with caloric restriction and no exercise, *WLe* obese rats treated with caloric restriction in combination with physical exercise, *WLsAM* obese rats treated with caloric restriction in combination with AM251 administration and no exercise, *WLeAM* obese rats treated with caloric restriction in combination with physical exercise and AM251 administration. Weight-maintenance interventions (WM) on weeks 16–21: *WMs* rats treated with diet SD and no exercise, *WMe* rats treated with diet SD in combination with physical exercise, *WMsAM* rats treated with diet SD in combination with AM251 administration and no exercise, *WMeAM* rats treated with diet SD in combination with physical exercise and AM251 administration.

Gene expression of transcripts related to vascular adhesion and angiogenesis (*Vcam, Sele, Vegfa*) were higher in the aorta of HFD vs SD controls after 21 weeks of the experimental period (Fig. 3A). In contrast, the different weight-loss and maintenance interventions caused a significant decrease in expression levels to values lower than those observed in SD. Such changes were matched by significant improvements in plasma atherogenic index (Fig. 3B). Likewise, gene expression of *Nos2*, related to the inflammatory status, was higher in the aorta of HFD-fed animals and this increment was reversed by weight control interventions that also down-regulated *Ptgs2* expression.

### Obesity-induced alterations in renal function and antioxidant capacity are reverted by the weight-loss and maintenance strategies implemented

Creatinine clearance ratio was significantly increased under our experimental conditions by DIO and AM251 administration during the first 2 stages of the experimental period (Table 3). Also, the obesity-related increase was associated with incipient albuminuria at 15 weeks that became significant at 21 weeks (HFD vs SD). Such was not the case with AM251 administration in which no albuminuria was detected. Creatinine clearance ratio also appeared to be affected by age of the animals and increased in SD rats at 21 vs 12 or 15 weeks. In contrast, caloric restriction and physical exercise tended to normalize all the above markers of altered renal functionality although results did not reach statistical significance in all cases.

**Table 3:** Effects of DIO and weight control interventions on urinary parameters of kidney functionality.

	Urinary volume (mL)	Urinary pH	Phosphorus (mg/dL)	Calcium (mg/dL)	Albumin (g/dL)	Creatinine (mg/dL)	Renal clearance (ml/min)
**12 weeks**							
SD	6.25	7.86	2.22	12.08	0.081	89.7	1.53
HFD	4.33	6.10	88.9***	3.15***	0.062	94.2	3.76*
SEM	0.78	0.160	0.048	0.232	0.014	3.69	0.53
**15 weeks**							
SD	5.99a	8.33c	2.31a	16.8b	0.077bc	79.50a	1.33ab
HFD	4.15a	6.73abc	88.8c	2.51a	0.112c	103.3b	2.55bc
WLs	5.37a	7.58bc	63.2bc	1.56a	0.061abc	88.4ab	1.41ab
WLe	5.26a	7.35bc	45.7b	2.43a	0.042ab	92.4ab	0.932a
WLsAM	13.2b	6.38ab	8.32a	8.95ab	0.012a	89.1ab	4.49d
WLeAM	12.1b	5.21a	3.60a	16.2b	0.034ab	77.6a	3.84 cd
SEM	1.11	0.838	10.32	2.711	0.017	7.07	0.47
**21 weeks**							
SD	7.06b	6.79ab	81.2c	1.73a	0.042a	95.1a	3.90a
HFD	4.20a	6.45a	95.9c	4.89ab	0.168b	101.5a	3.60a
WMs	8.07b	7.98b	2.46a	13.5c	0.043a	88.1a	1.77a
WMe	7.83b	8.26b	2.48a	7.09abc	0.071ab	94.9a	1.94a
WMsAM	14.9c	8.20b	4.32a	10.9bc	0.057a	91.7a	3.65a
WMeAM	13.2c	6.42a	64.3b	9.54abc	0.062a	93.7a	4.10a
SEM	1.34	0.550	5.41	2.78	0.032	6.58	0.841

*SD* standard rat chow diet, *HFD* hypercaloric diet for dietary induction of obesity, *WLs* high protein weight-loss intervention diet with a sedentary lifestyle (weeks 12–15), *WLe* high protein weight-loss intervention diet with training protocol, *WLsAM* high protein weight-loss intervention diet with a sedentary lifestyle and pharmacological treatment with AM251, *WLeAM* high protein weight-loss intervention diet with training protocol and pharmacological treatment with AM251. *WMs* high protein weight-loss intervention diet with a sedentary lifestyle (weeks 12–15) followed by weight-maintenance stage (weeks 15–21) with SD dietary treatment and sedentary lifestyle, *WMe* high protein weight-loss intervention diet with training protocol followed by weight-maintenance stage with SD dietary treatment and training protocol, *WMsAM* high protein weight-loss intervention diet with a sedentary lifestyle and pharmacological treatment with AM251 followed by weight-maintenance stage with SD dietary treatment, sedentary lifestyle, and pharmacological treatment with AM251, *WMeAM* high protein weight-loss intervention diet with training protocol and pharmacological treatment with AM251 followed by weight-maintenance stage with SD dietary treatment, training protocol, and pharmacological treatment with AM251. Results are means of 8 rats. SEM, standard error of the mean. *P < 0.05, ***P < 0.001 in t-test (12 weeks); a, b, c, means within the same column with different letters are significantly different (ANOVA treatment, P < 0.05; 15 and 21 weeks).

Regarding the urinary volume and parameters related to kidney stone formation, obesity induction led to a considerable decrease in urinary volume and pH as well as increased phosphaturia at weeks 12 and 15, which run in parallel to a decreased calciuria and increased calcemia (Table 2). The metabolic status of both minerals tended to be normalized by the weight control strategies implemented. Renal functionality was also affected by age of the animals, and considerable changes in urinary pH, phosphaturia, calciuria, and renal clearance were evident in the rats fed the SD diet on week 21 compared to weeks 12 and 15.

To assess whether obesity-induced changes in renal functionality could be mediated through increased oxidative stress, the kidney activity of antioxidant enzymes and lipid peroxidation were assessed (Table 4). Results are complex and numerous interactions are observable among obesity and weight-loss interventions on the former parameters. Consumption of HFD worsened oxidative stress conditions and resulted in higher Mn-SOD and GPX activities in all the stages of the experimental period, whereas Cu/Zn-SOD activity exhibited an HFD-derived increase on week 15 and a reduction on week 21. The effects of weight control interventions differed based on their implementation during the 3 weeks of weight-loss or the 6 weeks of weight-maintenance.

Specifically, on week 21 of experimental period, there was a significant decrease in Mn-SOD activity caused by the combined action of exercise and AM251 administration. Likewise, a marked decrease was also found in GPX activity caused by the administration of AM251, alone or in combination with physical exercise.

**Table 4:** Effects of DIO and weight control interventions on kidney antioxidant activities and lipid peroxidation.

	Mn-SOD (UAA/mg/protein)	Cu/Zn-SOD (UAA/mg/protein)	CAT (U/mg/protein)	GPX (nmol NADPH/min/mg protein)	TBARs (nmol MDA/mg protein)
**12 weeks**					
SD	72.9	583.4	18.1	22.7	1.63
HFD	89.8**	581.3	20.9	26.4	1.24**
SEM	4.75	35.3	4.21	4.23	0.42
**15 weeks**					
SD	35.1a	547.1b	13.9a	22.1a	0.71a
HFD	50.6b	785.1c	15.7ab	23.9a	1.33bc
WLs	56.6bc	569.4b	18.7bc	27.1ab	1.66c
Wle	61.2bcd	465.2ab	16.4abc	21.7a	1.47bc
WLsAM	65.7 cd	427.1a	19.8c	31.7b	1.42bc
WLeAM	71.1d	531.6ab	16.3abc	33.7b	0.99ab
SEM	4.20	37.1	1.28	2.32	0.07
**21 weeks**					
SD	55.5a	449.5ab	26.1c	24.1a	0.902a
HFD	85.6 cd	353.1a	19.6abc	36.7c	1.15a
WMs	69.1abc	433.5ab	24.7c	31.5bc	0.984a
WMe	98.4d	374.4ab	20.7bc	30.1b	1.39a
WMsAM	81.5bcd	419.3ab	12.9a	24.1a	1.36a
WMeAM	63.5ab	374.4ab	14.7ab	18.9a	0.913a
SEM	6.34	33.1	2.42	1.95	0.17

*SD* standard rat chow diet, *HFD* hypercaloric diet for dietary induction of obesity, *WLs* high protein weight-loss intervention diet with a sedentary lifestyle (weeks 12–15), *WLe* high protein weight-loss intervention diet with training protocol, *WLsAM* high protein weight-loss intervention diet with a sedentary lifestyle and pharmacological treatment with AM251, *WLeAM* high protein weight-loss intervention diet with training protocol and pharmacological treatment with AM251. *WMs* high protein weight-loss intervention diet with a sedentary lifestyle (weeks 12–15) followed by weight-maintenance stage (weeks 15–21) with SD dietary treatment and sedentary lifestyle, *WMe* high protein weight-loss intervention diet with training protocol followed by weight-maintenance stage with SD dietary treatment and training protocol, *WMsAM* high protein weight-loss intervention diet with a sedentary lifestyle and pharmacological treatment with AM251 followed by weight-maintenance stage with SD dietary treatment, sedentary lifestyle, and pharmacological treatment with AM251, *WMeAM* high protein weight-loss intervention diet with training protocol and pharmacological treatment with AM251 followed by weight-maintenance stage with SD dietary treatment, training protocol, and pharmacological treatment with AM251. *SOD* superoxide dismutase (Units/mg protein), *CAT* catalase (Units/mg protein), *GPX* glutathione peroxidase (nmol NADPH/min/mg protein), *TBARS* thiobarbituric acid reactive substances (nmol MDA/mg protein). Results are means of 8 rats. *SEM* standard error of the mean. **P < 0.01 in t-test (12 weeks); a, b, c, d, means within the same column with different letters are significantly different (ANOVA treatment, P < 0.05; 15 and 21 weeks).

## Discussion

The present study was carried out to assess the changes produced by obesity on some cardio-renal functions and to demonstrate the improvement in these functions due to a decrease in bodyweight and maintenance of lost weight. Caloric restriction, physical exercise, and blockade of CB1 receptor strategies were tested in an animal model of DIO.

Under our experimental conditions, obesity was successfully established (difference in body weight between normocaloric and HFD-fed animals was equal or greater than 2 standard deviations) from the 5th week of the DIO period by consuming an obesogenic diet compared to the normocaloric groups. Other models of DIO have been described^18,19^. However, it should be highlighted that this dietary combination led to rapid weight gain in the experimental animals. Once obesity was established, the HFD diet continued to be administered along all the stages of the experimental period to clearly show the related alterations. Afterward, during the 12–15-week intervention period, different weight-loss strategies were implemented that resulted in subsequent body weight loss. Such decrease can be a consequence of several interacting factors like the high protein content of the diet due to its thermogenic action and its high levels of satiating soluble dietary fiber, the potential anorexigenic action of physical exercise, the inhibitory action on food intake of AM251 at the hypothalamus and leptin sensitivity which is usually inhibited by the consumption of a high-fat diet^20–23^. In this context, administration of AM251 has been shown to display dose-dependent decreases in food intake and weight gain, specially at doses of 2–5 mg/kg^24^. In addition, the inhibitory action of AM251 can be potentiated by co-administration of leptin. Recently, a crosstalk between CB1 and GLP1 receptors has been described that provides new therapies for obesity^25^. The coadministration of a peripheral CB1 receptor inhibitor with long-acting GLP1R agonists achieves greater reduction in body weight and fat mass than monotherapies by promoting negative energy balance.

During 15–21 weeks, weight-maintenance strategies were combined revealing that the combination of the three interventions assayed: a certain degree of caloric restriction, physical exercise, and administration of CB1 receptor blocker was the most efficient to successfully maintain body weight and avoid the rebound effect common to other weight-loss treatments. In fact, during this last stage of the experimental period, the mixed training protocol and, especially, AM251 administration, contributed to maintain body weight via down-regulation of hypothalamic transcripts coding for Npy. Administration of AM251 has been reported to produce a significant decrease in the number of neurons expressing orexin A in the hypothalamus^26^, whereas Orexin-A represses satiety-inducing POMC neurons and contributes to obesity via stimulation of endocannabinoid signaling^27^. On the other hand, Di Marzo et al.^28^ reported that defective leptin signalling is associated with elevated hypothalamic levels of endocannabinoids in obese db/db and ob/ob mice and Zucker rats, whereas acute leptin treatment of normal rats and ob/ob mice reduces the endocannabinoids anandamide and 2-arachidonoyl glycerol in the hypothalamus.

DIO after consumption of HFD during 12 weeks resulted in the instauration of insulin resistance, as it is shown by the higher AUC after an oral glucose overload compared to SD groups Nevertheless, this pathological situation was reversed by weight loss interventions and AUC returned to values similar to those of SD animals. Other strategies including legume-derived functional ingredients and/or physical exercise^29^ have also confirmed this beneficial action. Lost weight maintenance further improved the glycemic profile of the animals after the combined action of physical exercise and AM251 administration, enhancing metabolic activities that promote insulin sensitivity.

The CB1 receptor is highly expressed in central and peripheral nervous system, as well as various peripheral tissues^30,31^. Evidence suggests that endocannabinoids are directly involved in the control of food intake and energy utilization by targeting these central and peripheral sites, including skeletal muscle, liver, and adipose tissue^32^. The effects of AM251 on glucose metabolism can be explained in a similar way to other CB1 receptor inverse agonists^33^ via metabolic “peripheral” action in adipose tissue in addition to its known “central” effect on food intake, thus providing an effective strategy for improving insulin sensitivity. The observed beneficial action of AM251 is also in agreement with the results of Esposito et al.^34^, who concluded that modulation of CB1 receptor regulated uptake at the level of the PI3K signaling system in skeletal muscle cells. Therefore, interfering with CB1 signaling could ameliorate glucoregulatory functions in peripheral tissues. In a similar way, Crespillo et al.^35^ suggested that blockade of CB1 receptor could play an important role in the restoration of compromised metabolic status caused by high fat intake and improve cardiometabolic risk factors. Furthermore, in a recent study, Eid et al.^36^ have tested the effects of two neutral CB1 receptor antagonists (central and peripheral) with improved safety profiles on a preclinical model of insulin resistance. Both compounds alleviated insulin resistance peripherally, and exerted similar effects on rats with metabolic syndrome. They also displayed anti-dyslipidemic, anti-hyperuricemic and anti-inflammatory effects.

Consumption of a diet rich in saturated fats has been associated with changes in heart weight running in parallel to the development of obesity and insulin resistance and may cause ventricular modifications, increasing left ventricle mass that will, in turn, lead to diastolic and systolic alterations and modified left ventricle ejection ratio^37^. Those changes are reflected in electrocardiographic modifications like the increased amplitude of QRS complex or QTc interval related to cardiac pathology and ventricular arrhythmia^38^. Here, HFD-induced alterations in cardiac functionality appeared to be mostly related to the degree of cardiac hypertrophy and took place mainly at the ventricular level, resulting in the higher amplitude of QRS complex and T-wave as well as lengthening of the corrected QT interval. Pathological alteration of these parameters indicates disturbances in the electrical activity of the heart and, consequently, on the efficiency of pumping blood. Here, such changes began to show a trend at 12 weeks and were significant at 15 weeks. We have not observed any changes in ECG parameters related to atrial depolarization and conduction (P wavelength, P wave amplitude, or PR interval), reinforcing the idea that obesity affected mainly ventricular function. Modifications in ECG (QRS amplitude and T wave amplitude) were corrected after the weight loss interventions, although no additional effect to that of caloric restriction was achieved by exercise, CB1 receptor blockade, or the combination of both interventions. Furthermore, no significant effect of exercise was found on QTc interval in a similar way to what has been reported in the obese Zucker rat model^39^. The improvement in parameters of cardiovascular health achieved by the weight-loss interventions and weight-maintenance was closely associated with significantly higher aerobic capacity and markers of physical fitness like maximum speed and total distance run during an incremental test. AM251 showed a very strong enhancing action on the later parameters in agreement with Zhou and Shearman^40^ who described a similar action of AM251 administration and physical activity on body weight loss and aerobic capacity. In contrast, Zhou and Kumar^41^ stated that inhibition of CB1 receptors could have a negative influence on the practice of physical exercise. The benefits of AM251 can be attributed to the role played by CB1 receptors on skeletal muscle metabolism. Their blockade using the inverse agonist SR141716 has been described to induce a greater entrance of glucose from the bloodstream to muscle cells and potentially greater storage of energy during the practice of physical exercise, which, in turn, is consistent with the improvement in glucose AUC^42^.

An important factor to be considered related to cardiac function is vascular functionality. In this regard, obesity, MetS, and lack of physical activity may cause endothelial dysfunction and early vascular ageing^43,44^ by over-expression of vascular adhesion molecules associated with atherogenesis manifested before necrosis and coronary lesions. In our study, the increased aerobic capacity was associated with improved vascular health assessed by the aorta expression of different transcripts related to endothelial adhesion molecules (*Sele* and *Vcam*) and inflammation parameters (*Nos2*, *Ptg2*, *Vegfa*) at the end of the 21-week experimental period. Normalization of caloric intake as well as the mixed training protocol significantly down-regulated their expression, reaching values even lower than those of the SD group, and reduced the plasma atherogenic index, a widely used marker of dyslipidemia, obesity, and cardiovascular disease^45,46^. Altogether, these results show a strong positive response to our weight loss and maintenance interventions at a cardiovascular level.

Obesity also represents a key risk factor for kidney disease. The consumption of HFD in our study caused a marked increase in their renal clearance rate, a measure of glomerular filtration, associated with albuminuria, a well-known marker of renal damage, both being reversed by the weight-loss and maintenance interventions of caloric restriction and physical exercise to values similar to SD group. Pharmacological treatment with specific CB1 receptor antagonist has been described to improve renal structure and functionality in experimental models of chronic kidney disease^47^. Moreover, a recent study by Udi et al.^48^ describes the administration of a novel peripherally restricted, orally bioavailable dual CB1 receptor/iNOS antagonist on a preclinical model of obesity-induced chronic kidney disease. The hybrid inhibitor ameliorated obesity-induced kidney morphological and functional changes via decreasing kidney inflammation, fibrosis, oxidative stress, and renal injury. Interestingly, some of these features were independent of the improved metabolic profile induced by the inhibition of CB1 receptors. Here, AM251 administration contributed strongly to the weight loss strategy, helped to reverse the obesity-induced alterations in glucose metabolism and caused a significant increase in glomerular filtration rate and diuresis. However, in a different way to what was observed in animals fed the HFD, it did not correlate with the presence of albuminuria. In this regard, Jenkin et al.^47^ reported that chronic administration of AM251 for 6 weeks with a daily dose of 3 mg/kg significantly reduced weight gain, systolic blood pressure, plasma leptin, albuminuria, plasma creatinine, and tubular cross-section diameter in a DIO model of Sprague Dawley rats, whereas Barutta et al.^49^ have reported that blockade of CB1 receptors ameliorates albuminuria in a diabetic nephropathy mice experimental model. Under our experimental conditions, the decreased albuminuria could also be due to a dilution effect associated with the higher diuresis in AM251-administered rats.

The increase of glomerular filtration rate resulting from AM251 administration is in agreement to what has been reported by Koura et al.^50^. Furthermore, Sampaio et al.^51^ have described the inhibitory action of the former compound in Na/k-ATPase activity of tubular cells. This would in turn decrease Na reabsorbtion and increase natriuresis, increasing urinary volume as observed in AM251-administered rats of the present study. On the other hand, obese rats of our experiment showed clear signs related to Parathormone activation at 12 or 15 weeks of experimental period, characterized by increased phosphaturia and decreased calciuria. AM251 administration reversed such effects independently of weight loss after 3 weeks of AM251 treatment.

Alterations in renal antioxidant status induced by obesity are known to contribute to renal damage^52^. DIO resulted in clear signs of kidney oxidative stress characterized by altered Mn and Cu/Zn-SOD and, to a lower extent, GPX activities, whereas the normalizing effects of weight control interventions were only relevant in Cu/Zn-SOD status. Blockade of the CB1 receptor has been shown to protect against tubular damage attenuating renal oxidative stress and inflammation^53^. Under our experimental conditions, administration of AM251 was effective at normalizing the status of Mn-SOD and GPX activity at week 21.

In conclusion, weight-loss and maintenance intervention strategies tested were efficient at reversing the obesity-related alterations in body weight, aerobic capacity, glucose metabolism, cardiovascular and renal function. The beneficial action was very consistent for caloric restriction and physical exercise, whereas AM251 administration complemented their effects on parameters like body weight and exhibited significant benefits on aerobic capacity. The implementation of weight control strategies in overweight or obese subjects that can avoid the post-treatment rebound effects are very effective measures to revert the metabolic, cardiovascular, and renal alterations developed in parallel to caloric imbalance, sedentarism, and obesity.

## Material and methods

### Animals and experimental design

The selection of the right in vivo experimental model is a crucial point in obesity studies. The Sprague Dawley rat is an adequate animal model of diet-induced obesity (DIO) as it develops obesity, dyslipidemia, insulin resistance, hypertriglyceridemia, and altered cardiovascular and renal functionality after the intake of a high-fat diet (HFD) for a period of 8–12 weeks^54^. The experiment used 112 male Sprague Dawley rats with an average body weight of 184 ± 10 g (6-weeks old, Charles Rives, Barcelona, Spain) that were allocated into fourteen different experimental groups (n = 8/group). The animals were housed in a well-ventilated, thermostatically controlled room (21 ± 2 °C) (Unidad de Experimentación Animal, CIC, Universidad de Granada). A reversed 12:12 light/dark cycle was implemented so that AM251 administration and the training protocol would be performed in darkness. Throughout the trial, animals had free access to type 2 water (resistivity 15 MΩ^−cm^) and consumed the diet ad libitum*,* except the intervention groups in the weight-maintenance stage of the experiment that were adapted to slightly lower food intake.

The experiments lasted for 21 weeks and included three different stages: (i) obesity induction and related metabolic alterations 0–12th week, (ii) weight-loss intervention 12th–15th week and (iii) weight maintenance intervention 15th–21st week) (Fig. 1A).

Throughout the experimental period, a standard normocaloric group was used as a control diet, SD (Teklad Global Diet 2014; 2.4 kcal/g) for the different stages. Obesity was induced using a hypercaloric obesogenic diet, HFD (including 60% of kcal as fat, Research diets D12492; 5.2 kcal/g), for 21 weeks. The weight-loss intervention stage (WL) included a caloric restriction strategy based on a reduction in the amount of food consumed and using a high protein and soluble fiber diet (2.5 kcal/g, Table 5) with/without physical exercise intervention, for 3 weeks and/or administration of AM251 inhibitor. Finally, the experiments continued with a weight-maintenance intervention (WM) that was achieved by the inclusion of a standard normocaloric diet, for 6 weeks, *pair fed* to 23 g/days to achieve a 12–15% caloric reduction compared to the same period in control SD group as part of the strategy to maintain the lost weight during maintenance stage avoiding the rebound effect^55^ with/without physical exercise intervention, and/or administration of AM251 inhibitor.

**Table 5:** Nutrient composition of the experimental diets.

	SD	HFD	WL
Protein (%)	13.5	22.2	26.7
Ash (%)	4.53	4.11	4.2
Fiber (%)	22.3	5.0	23.1
Carbohydrate (by difference) (%)	55.3	37.5	41.8
Energy (Kcal/g)	2.4	5.2	2.5
Fat (%)	4.41	31.2	4.23
**Fatty acid profile (%)**			
Miristic (C14:0)	–	0.25	–
Palmitic (C16:0)	10.36	21.33	7.20
Stearic (C18:0)	0.46	7.10	1.30
Palmitoleic (C16:1n9)	–	0.36	–
Oleic (C18:1n9)	12.84	41.62	78.16
Octadecenoic (C18:1n7)	–	0.36	–
Linoleic (C18:2n6)	74.35	27.86	12.42
Linolenic (C18:3n3)	1.99	1.13	0.92

*SD* standard rat chow, *HFD* hypercaloric diet for dietary induction of obesity, *WL* high protein intervention diet for weight loss. Formulation of WL diet per 100 g: pea protein isolate 21%, whey protein isolate 18%, cysteine 0.2%, olive oil 4%, Cellulose 11%, *Plantago ovata* fiber 11%, AIN93M mineral premix 3.5%, AIN93M vitamin premix 1%, coline bitartrate 0.25%, dextrins 15%, wheat starch 15%.

The administration of AM251 was carried out by intraperitoneal injection thrice a week (Monday, Wednesday, Friday at 9:00 a.m.) at a dose of 3 mg/kg body weight^56^ during the 3-week weight-loss intervention (WL, 12–15 weeks) and once a week during the maintenance period (WM, 16–21 weeks).

The weight-loss intervention and bodyweight maintenance periods have been designed based on the information provided by Sengupta^57^ who reported that laboratory rats live about 2–3.5 years (average 3 years), while the worldwide life expectancy of humans is 80 years. Thus, one human year almost equals two rat weeks (13.8 rat days) when correlating their entire life span. Under our experimental conditions, 3 weeks equal to 1.5 years of human life, which is a long enough period to achieve an efficient weight loss. The maintenance period of 6 weeks equals 3 years which is enough to demonstrate the success of our combined strategy against bodyweight-regain.

The composition of different experimental diets is shown in Table 5.

The diet was provided for all four animals in each cage but the bodyweight control was registered individually. Caloric intake was recorded daily whereas body weight was measured once a week. During the last week of each experimental stage, a 12 h fasting urine sample collection was performed for each animal located during that time in individual metabolic cages designed to achieve a separate collection of feces and urine. Volume, pH, and other biochemical parameters were then measured in urine. Moreover, a glucose overload tolerance test was performed 48 h after the last training session. The animals were allowed to recover for 48 h before fasting for a further 8 h, followed by anesthesia with ketamine (75 mg/kg body weight) and xylazine (10 mg/kg body weight) and electrocardiogram recording before sacrifice by exanguination. Blood was collected by puncture of the abdominal aorta (with heparin as an anticoagulant). An aliquot of 0.25 mL was used to assess blood parameters (KX-21 Automated Hematology Analyzer, Sysmex Corporation), and the rest was centrifuged at 1458×*g* for 15 min to separate plasma that was subsequently frozen in liquid nitrogen and stored at – 80 °C^58^. The kidney and heart were extracted, weighed, and immediately frozen in liquid nitrogen and stored at − 80 °C until they were processed for antioxidant activity assessment and RNA extraction. The whole hypothalamus and descending aorta were extracted, immersed in RNA preserving solution (RNAlater, Ambion), and stored frozen until RNA extraction. The femur was extracted and its length was measured with a caliper before freezing in liquid nitrogen and storage at − 80 °C. All experiments were undertaken according to Directional Guides Related to Animal Housing and Care^59^ and all procedures were approved by the Animal Experimentation Ethics Committee of the University of Granada, Spain (Project Reference DEP2014-58296R).

### Training protocol

Rats trained following a protocol based on interval aerobic training combined with strength exercise in the same session^60,61^. The animals run on a specially designed treadmill (Panlab, LE 8710R) and all sessions were performed 5 days/week, during the dark cycle of the animals. A final incremental test following the protocol described by Wisløff et al.^62^ and Clemente et al.^63^ was performed 96 h before the end of the weight-maintenance stage to test the maximal aerobic capacity and physical performance achieved by the animals as a result of the interventions.

### Intraperitoneal administration of AM251

The CB1 receptor inverse agonist, *N*-(Piperidin-1-yl)-5 (4-iodophenyl)-1-(2,4-dichlorophenyl)-4-methyl-1H-pyrazole-3-carboxamide (AM251) (Tocris Cookson, UK) was dissolved in the vehicle dimethyl sulfoxide:tween-80:0.9% NaCl (1:2:97) for intraperitoneal administration in rats at a dose of 3 mg/kg body weight^58^.

### Determination of body weight to femur length ratio

Bodyweight and femur length of the rats in all the control and experimental groups were measured at the end of every experimental stage (12, 15, and 21 weeks). Bodyweight to femur length ratio was determined by dividing body weight (g) by the square of femur length (cm) as a representative measure of the animal’s nose-anus length.

### Blood, plasma, and urine biochemical analysis

Fasting blood glucose and glucose tolerance tests were performed by oral glucose overload as previously described^64^ using a dose of 6.9 µmol glucose per gram body weight. The area under the curve (AUC) was calculated following the trapezoidal rule^65^. Plasma and urinary biochemical parameters were analyzed using a Shenzhen Midray BS-200 Chemistry Analyzer (Bio-Medical Electronics). Analytical kits (Spin-React, Spain) were used to measure the following parameters in plasma [total-cholesterol, HDL-cholesterol, uric acid, urea, creatinine, total protein, albumin, Ca, P, creatine kinase MB (CK-MB), and angiotensin-converting enzyme activity (ACE)], and urine [total protein, albumin, creatinine]. Plasma atherogenic Index (AI) was calculated with the formula: AI = (total cholesterol − HDL-cholesterol)/HDL-cholesterol. Urinary pH was analyzed using a bench pH-meter. Renal creatinine clearance rate as an index of glomerular filtration rate was calculated using the standard formula C = U × V/P, where U is the concentration in urine (mg/dL), V is the urine flow rate (mL/min), and P is the plasma concentration (mg/dL).

### Electrocardiogram recording

ECG was recorded as previously described^66^ with surface electrodes for 1 min using the LabChart 7 software (AD Instruments, USA) at the end of the experimental period. The average value for each rat was obtained from values of ten consecutive cardiac cycles measured at 10, 30, and 50 s. The following parameters were measured: P-wave length and amplitude, PQ interval, QRS complex length and amplitude, T-wave length and amplitude, and QT interval. Corrected QT interval (QTc) was calculated using Bazett’s Formula (QTc = QT interval/√(RR interval).

### RNA extraction and quantitative RT-PCR

Total RNA was isolated from the whole hypothalamus and the descending aorta by homogenization in 1 mL of Tri-Reagent (Sigma-Aldrich). The RNA was solubilized in Rnase-free H_2_O and treated with DNase (Applied Biosystems) to remove any DNA present in the sample. A total of 100–250 ng of RNA was reverse-transcribed according to standard protocols using a Lifepro Thermal Cycler (Bioer Serves Life, China). Quantitative RT-PCR was performed with 7900 HT Fast Real-Time PCR system (Applied Biosystems), using primer/probes for genes involved in food intake and energy metabolism in the hypothalamus [*fos, Npy, Hcrt, Lepr, Cnr*] or genes coding for adhesion proteins and enzymes involved in inflammatory pathways in the descending aorta [*Vcam, Sele, Vegfa, Nos2, Ptgs2*] (Applied Biosystems). The PCR master mix reaction included the first-strand cDNA template, primers/probes, and 2X TaqMan Fast Universal PCR Master Mix, No AmpErase UNG (Applied Biosystems). Relative quantification was performed using the comparative Ct (2 − ΔΔCt) method^67^. ß-actin was used as internal control.

### Kidney antioxidant activity and lipid peroxidation assays

A fresh kidney aliquot was homogenized (1:10 w/v) in 50 mM phosphate buffer (pH 7.8) containing 0.1% Triton X-100 and 1.34 mM of DETAPAC. Kidney homogenates were centrifuged at 13,000×*g*, 4 °C for 45 min, and the supernatant was used to determine the activity of antioxidant enzymes. Catalase activity was assayed according to Cohen et al.^68^, total cellular Glutathione Peroxidase (GPX) activity by the coupled assay of NADPH oxidation^69^ using cumene hydroperoxide as substrate, and total superoxide dismutase (SOD) activity following the methodology of Ukeda et al.^70^. Mn-SOD activity was determined by the same method after treating the samples with 4 mM KCN for 30 min. Cu/Zn-SOD activity resulted from the subtraction of Mn-SOD activity from the total SOD activity. Protein concentration was assayed by the method of Bradford^71^. Lipid peroxidation was determined in kidney homogenates according to the methodology of Ohkawa et al.^72^. Thiobarbituric acid reactive substances (TBARS) were assayed spectrophotometrically at 532 nm.

### Statistical analyses

Significant differences in aerobic capacity, glycemic profile, plasma, and urinary biochemical parameters and indices, electrocardiographic parameters, gene expression data, and kidney antioxidant enzyme activity data between SD and HFD groups were analyzed by t-test at the end of DIO stage (12 weeks). At the end of weight-loss (WL, 13–15 weeks) and weight-maintenance (WM, 16–21 weeks) stages, significant differences were analyzed by one-way ANOVA among all experimental groups in each stage. Tukey’s test was used to detect differences between treatment means. Statistical analysis was performed with Statistical Package for Social Sciences (IBM SPSS for Windows, version 22.0, Armonk, NY), and the level of significance was set at p < 0.05.
